# Opinions of Foreigners Respecting English Medical Politics

**Published:** 1830-04-01

**Authors:** 


### OPINION OF ENGLISH MEDICAL POLITICS ENTERTAINED BY FOREIGNERS.

Men of all parties, and in all countries, have looked with great curiosity towards the opinions entertained of them by strangers, by foreigners who could have no bias in favour of one sect or party rather than of another. This curiosity is no doubt strengthened by the consciousness that, if justice or candour is to be found at all, it is more likely to come through a neutral channel than in any other way. Our transatlantic brethren cannot possibly have any feeling in favour of any party in the body politic of medicine here—or if there should be any leaning at all, it must, for obvious reasons, be towards the democratic part of the profession. Let us hear what they say and think of us at a distance of three thousand miles from our modern Babylon. The following extracts are from a long and extremely well-written article, in the Boston Medical and Surgical Journal, for July 1828.

After alluding to the great number of medical men collected together in this metropolis, and the necessary or unavoidable collisions of interest or rivalry that must occasionally take place, the Editor goes on thus :—

" Such a community can never be without its dissensions, contests, and hostilities. Still as these were not alluded to in the public journals, they were unknown beyond the limits of the city, and probably of the hospital or district in which they existed. Within a few years, a new state of things has arisen. A journal was established—the Lancet—the proposed object of which was Medical and Surgical Reform. Abuses, it was asserted, had crept into the management of medical institutions—monopolies existed—ignorance and incompetence were in places of trust and honour—' preferment went by favour and affection.' The Editor of the Lancet was to perform the Herculean task of cleansing away the augean filth which had accumulated. He established himself as the censor of the medical world; employed himself in prying into the intimate causes of all the evils he pointed out, and was to bring to light and expose in open day, all who in any way were interested in, or promoted the abuses which existed.

" That abuses did exist, that the profession have been roused to a sense of their pernicious influence, and that effective steps have been taken towards their remedy, cannot be doubted ; neither, we think, can it be well denied that the influence of the Lancet, in this particular, has been beneficial. Neither can it be doubted that, by keeping a watch over the conduct of the individuals having charge of the public institutions, it has awakened them to a more thorough performance of their duty. The knowledge that even inadvertent and unavoidable errors would be exposed and dwelt upon with scrupulous malignity, must make such persons particularly cautious and attentive in the performance of their public duties. Besides, by the publication, in the Lancet, of Lectures of teachers and of the hospital reports of the whole city, unfair as they often have been, and coloured and distorted for particular purposes, a great mass of valuable professional matter has been circulated; and what is better, the fashion of publication has been set, which will be followed to the manifest advantage of the science.

" It is right to give credit where it is due, and it must be admitted that, to this extent the influence of the Lancet has been beneficial. Yet we doubt whether even this much has proceeded from the pure desire of doing good. The temper, spirit and motive of this journal appears to be radically bad. It is vulgar, low and abusive. If it cries down abuses, it seems actuated more by a hatred of those who benefit by them, than from any proper love of right and justice. Its malignity has been chiefly directed against what may be called the aristocracy of the profession; that is to say, against those individuals, who, by their talents, opportunities, patronage, or any other causes, have become eminent in public opinion as professional men. The characters and conduct of these individuals are attacked without reserve, and assailed in the most indecent language. Their practice at the hospitals, their operations, their writings, are criticised unsparingly. And, what is worse than all, those unfortunate and unsuccessful occurrences in practice, which occasionally fall to the lot of every practitioner, are seized upon with avidity, dragged out to public notice, represented in the most unfavourable light—the practitioner accused of ignorance and want of skill, and held up as an object worthy of public indignation.

"It will not be amiss we think to present to our readers a few examples of the style in which these attacks are made. It will be instructive, and may serve to warn us from giving in, in any degree, to that spirit of jealousy and detraction which has been said to be characteristic of our profession. It is also an important fact in the medical history of our times, that a journal conducted in such a spirit, should have acquired and maintained so extensive a popularity, and exercised so great an influence in the most enlightened city of the world. Many in the profession have, as it appears, actually stood in awe of this common libeller, for all who have had the independence and spirit to stand out in open hostility, have been immediately made the subjects of its attacks."

The editor goes on to give a great variety of samples of eloquence from the Lancet, as directed against Dr. M'Leod, Mr. Travers, Mr. Earl, Mr. Brodie, Mr. Bransby Cooper, and others—overlooking, we know not why, the delectable morsels of abuse that have been so often showered upon our own heads. He then remarks .—

"We do not introduce these quotations to amuse our readers, or to gratify that propensity for listening to slanderous tales, which exists too strongly, even in the best of men; but for the purpose of displaying the temper and spirit of which we have spoken. Even the rival journal, the London Medical Gazette, although professedly established for the purpose of putting down this temper, has, sometimes, not a little given in to it. Mr. Lawrence is the surgical hero of the Lancet, who can do nothing wrong. Mr. Lawrence accordingly becomes the object, not indeed of thorough Lancet-like abuse, but of something approaching it, in the columns of the Gazette."

Mr. Bransby Cooper's operation is next taken up, for the trial had not then taken place, and the statement in the Lancet, even when there was but one side of the case before the public, is treated with feeling of just indignation.

"But the most gross violation of decency, and of offence against manly and generous feeling, by the Lancet, occurs in the account which it gives of a late unfortunate operation for stone by a nephew of Sir Astley Cooper at Guy's Hospital. This account, as it made a great sensation in the medical world, and is likely to be made the subject of a judicial investigation, we quote entire."

Then follows the theatrical representation enacted by manager Lambert, with the American commentary.

"Whatever may have been the faults of the operation, or the blunders of the surgeon, no man of proper feelings can regard with any thing but unqualified disgust, the air of malignant triumph with which this unhappy case is dwelt upon; and the various measures taken by the students who witnessed the transaction, and by other surgeons, indicate a strong sympathy with Mr. Cooper, and a just indignation against his libeller."

"But to proceed no farther in this humiliating exposition, we have only to add as evidence of the state of things produced by this publication, that within less than a year three suits at law and a duel have been brought about between medical men wholly by the influence of the Lancet. Now whatever good ends may have been held in view and attained, the means must be bad which lead even indirectly to such consequences as these.

"Yet we believe, not only as a matter of duty, but *even of policy*, that it is wise to cultivate kind and friendly feelings towards those who come most directly across our paths, and always to speak of them in the most favorable terms that strict regard to truth will allow; never to take advantage of those accidents and adverse occurrences which happen in practice to the most accomplished and successful men, to create prejudice against others, but on the contrary distinctly to impress the opinion, that from the nature of the art such occurrences are inevitable. We believe that those who candidly and honestly pursue this course will find their account in it. It will produce not only more kindly feelings towards them, and a greater confidence in them among their professional brethren, but a more desirable and permanent reputation in the public at large; and afford them a degree of happiness, which success, with an opposite temper, could not give.

"We sincerely believe that no such spirit now exists in this country as is evinced in the publications we have been speaking of. But it by no means follows that such a state of things may not arise, and it becomes those who are looked up to on account of their age or eminence, and who have a proper regard to the honor of their profession, to set an example of candor, moderation and liberality, which cannot fail to have the most salutary effects."

Every honorable man who has the interest of his profession at heart, or who is imbued with any of the better feelings of humanity, will join in the excellent advice and wise precepts of our transatlantic contemporary, whose sentiments do honour to his country as well as to himself. It may be gratifying to him to know that the influence of the "common libeller" is gone for ever, in this country—and well that libeller knows it! The hallucination is passing fast away from the medical profession of these Isles, though not before its members became the subject of astonishment, and too often of contempt, among their brethren in other countries. The violence of the infatuation has soon exhausted, and consequently cured itself—and the instrument which once created awe among the timid, is now looked upon with as much indifference, if looked upon at all, as the log thrown down by Jupiter was eyed by the croaking tribe, after the splashings had subsided.

